# Supplementary material for: MetaRibo-Seq measures translation in microbiomes
Source: Nat Commun. 2020 Jun 29;11:3268. doi: 10.1038/s41467-020-17081-z (PMC7324362; doi:10.1038/s41467-020-17081-z)
Supplement: Supplementary file 10 — Supplementary Data 7 [file 41467_2020_17081_MOESM10_ESM.zip › File2/Confidence_High_Taxonomy/261137_out.krona.html]

Javascript must be enabled to view this page.

members
magnitude
magnitudeUnassigned
count
unassigned
taxon
rank

261137\_out

55

55
2
superkingdom

55
976
phylum

class
200643
55

171549
order
55

45
family
2005525

45
375288
genus


SRS019496\_contig\_number\_13742
1262912
species
1

1

SRS015782\_contig\_number\_contig-100\_432.254948
species
469591


SRS016095\_contig\_number\_14446SRS016989\_contig\_number\_16219SRS045195\_contig\_number\_6176SRS049712\_contig\_number\_24772SRS049900\_contig\_number\_3924SRS052027\_contig\_number\_12168SRS064645\_contig\_number\_1251SRS065397\_contig\_number\_4569SRS076929\_contig\_number\_26351SRS100021\_contig\_number\_8905SRS146813\_contig\_number\_4717
574930
species
11


SRS015217\_contig\_number\_21320SRS062654\_contig\_number\_15581SRS065504\_contig\_number\_contig-100\_196.193482SRS098644\_contig\_number\_contig-100\_532.262170SRS1041140\_contig\_number\_22213
46503
species
5


SRS015663\_contig\_number\_12649
2293123
species
1


SRS013638\_contig\_number\_6387SRS014459\_contig\_number\_23398SRS015065\_contig\_number\_34006SRS015431\_contig\_number\_contig-100\_130.487948SRS018836\_contig\_number\_14705SRS019068\_contig\_number\_contig-100\_91.650451SRS019397\_contig\_number\_12916SRS021219\_contig\_number\_11882SRS023583\_contig\_number\_contig-100\_152.88061SRS051031\_contig\_number\_24106SRS051610\_contig\_number\_8866SRS051882\_contig\_number\_22243SRS054590\_contig\_number\_220SRS058145\_contig\_number\_2396SRS062701\_contig\_number\_7218SRS075773\_contig\_number\_37163SRS078176\_contig\_number\_11530SRS1055038\_contig\_number\_531SRS1055069\_contig\_number\_9944SRS142712\_contig\_number\_1115SRS143876\_contig\_number\_40386SRS148196\_contig\_number\_contig-100\_327.242593SRS148511\_contig\_number\_11471SRS149244\_contig\_number\_9942SRS893231\_contig\_number\_1627
species
823
25


SRS075021\_contig\_number\_3033
species
563193
1

family
815
10

10
816
genus


SRS043667\_contig\_number\_contig-100\_406.41840SRS1055049\_contig\_number\_1673
821
species
2


SRS104693\_contig\_number\_11699
species
818
1


SRS016335\_contig\_number\_3568SRS047014\_contig\_number\_31739SRS142890\_contig\_number\_18568SRS143181\_contig\_number\_13841SRS148319\_contig\_number\_5186SRS893383\_contig\_number\_20953
820
species
6

1

SRS148874\_contig\_number\_1024
species
817
